# Ceratinadins E and F, New Bromotyrosine Alkaloids from an Okinawan Marine Sponge *Pseudoceratina* sp.

**DOI:** 10.3390/md16120463

**Published:** 2018-11-23

**Authors:** Shin-ichiro Kurimoto, Taito Ohno, Rei Hokari, Aki Ishiyama, Masato Iwatsuki, Satoshi Ōmura, Jun’ichi Kobayashi, Takaaki Kubota

**Affiliations:** 1 Showa Pharmaceutical University, 3-3165 Higashi-Tamagawagakuen, Machida, Tokyo 194-8543, Japan; kurimoto@ac.shoyaku.ac.jp (S.-i.K.); b13031@ug.shoyaku.ac.jp (T.O.); 2 Kitasato Institute for Life Sciences, Kitasato University, 5-9-1 Shirokane, Minato-ku, Tokyo 108-8641, Japan; hokari@lisci.kitasato-u.ac.jp (R.H.); ishiyama@lisci.kitasato-u.ac.jp (A.I.); iwatuki@lisci.kitasato-u.ac.jp (M.I.); omuras@insti.kitasato-u.ac.jp (S.Ō.); 3 Graduate School of Pharmaceutical Sciences, Hokkaido University, Sapporo 060-0812, Japan; jkobay@pharm.hokudai.ac.jp

**Keywords:** marine sponge, *Pseudoceratina* sp., bromotyrosine alkaloid, ceratinadin, 1,6-dioxa-2-azaspiro[4.6]undecane, 3,5-dibromotyramine, psammaplysin, moloka’iamine, antimalarial activity, *Plasmodium falciparum*

## Abstract

Two new bromotyrosine alkaloids, ceratinadins E (**1**) and F (**2**), were isolated from an Okinawan marine sponge *Pseudoceratina* sp. as well as a known bromotyrosine alkaloid, psammaplysin F (**3**). The gross structures of **1** and **2** were elucidated on the basis of spectroscopic data. The absolute configurations of **1** and **2** were assigned by comparison of the NMR and ECD data with those of a known related bromotyrosine alkaloid, psammaplysin A (**4**). Ceratinadins E (**1**) and F (**2**) are new bromotyrosine alkaloids possessing an 8,10-dibromo-9-methoxy-1,6-dioxa-2-azaspiro[4.6]undeca-2,7,9-trien-4-ol unit with two or three 11-*N*-methylmoloka’iamine units connected by carbonyl groups, respectively. Ceratinadin E (**1**) exhibited antimalarial activities against a drug-resistant and a drug-sensitive strains of *Plasmodium falciparum* (K1 and FCR3 strains, respectively).

## 1. Introduction

**Figure 1:**
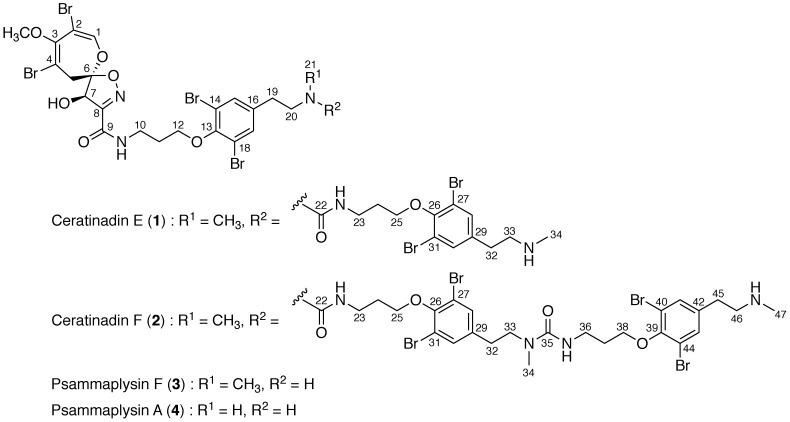
Structures of ceratinadins E (**1**) and F (**2**) and psammaplysins F (**3**) and A (**4**).

According to the World Health Organization (WHO), 216 million clinical cases of malaria occurred and 445,000 people died of malaria in 2016 [1]. The representative antimalarial natural products, quinine and artemisinin, and their derivatives have been widely used for the treatment of malaria. However, the therapeutic efficacy of these existing antimalarial drugs is being lost due to the emergence and spread of drug-resistant strains of malaria. Therefore, the development of a new class of antimalarial drugs is urgently needed. Marine sponges have been recognized as a rich source of unique bioactive natural products. A variety of bromotyrosine alkaloids with a wide range of biological activities have been isolated from marine Verongid sponges [2] and references therein. The bromotyrosine alkaloids possessing the 1,6-dioxa-2-azaspiro[4.6]undecane skeleton, such as pammaplysins F (**3**), G, and H, and 19-hydroxypsammaplysin E, have been reported to exhibit antimalarial activity [3,4,5,6] Notably, psammaplysins F (**3**) and G showed antimalarial activities not only against the drug-sensitive strain but also against the drug-resistant strain of *Plasmodium falciparum* [4]. During our search for new bioactive natural products from marine organisms, a series of bromotyrosine alkaloids has been isolated from Okinawan marine Verongid sponges [7,8,9,10,11]. Recently, we have isolated two new bromotyrosine alkaloids possessing the 1,6-dioxa-2-azaspiro[4.6]undecane skeleton, ceratinadins E (**1**) and F (**2**), see Figure 1, from the extract of an Okinawan marine sponge *Pseudoceratina* sp. Here, we describe the isolation, structure elucidation, and antimalarial activity of **1** and **2**.

## 2. Results

The marine sponge *Pseudoceratina* sp. collected at Okinawa, Japan, was extracted with MeOH, and the extract was partitioned between EtOAc and H_2_O. The new bromotyrosine alkaloids, ceratinadins E (**1**, 4.6 mg, 0.0073% wet weight) and F (**2**, 0.4 mg, 0.00063% wet weight), were purified from the EtOAc-soluble material by silica gel column chromatography, short C_18_ column chromatography, C_18_ flash column chromatography, and C_18_ HPLC. A known related bromotyrosine alkaloid, psammaplysin F (**3**, 10.8 mg, 0.017% wet weight) [3,4], was obtained as well as **1** and **2** from the EtOAc-soluble material.

**Figure 2:**
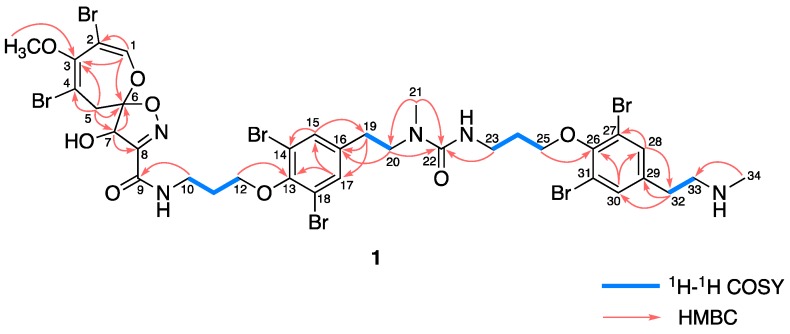
Selected 2D NMR correlations for ceratinadin E (**1**).

Ceratinadin E (**1**) was obtained as an optically active colorless amorphous solid. The ESIMS spectrum of **1** showed the pseudomolecular ion peaks at *m/z* 1133, 1135, 1137, 1139, 1141,1143, and 1145 (1:6:15:20:15:6:1, [M + H]^+^), indicating the presence of six bromine atoms, see Figures S1 and S2. The molecular formula of **1** was established as C_35_H_41_Br_6_N_5_O_8_ by HRESIMS data, see Figure S3. The UV absorption at 263 nm was attributed to the substituted benzenoid chromophore. The IR absorption at 3338 cm^−1^ implied the existence of hydroxy and/or amino groups, while the IR absorption at 1671 cm^−1^ implied the existence of carbonyl groups. The inspection of the HSQC spectrum with the ^1^H and ^13^C NMR data disclosed that **1** consists of fifteen non-protonated carbons, five sp^2^ methines, one sp^3^ methine, eleven sp^3^ methylenes, and three methyls, see Table 1 and Figures S4, S5, and S7. The comparison of the ^1^H and ^13^C NMR data of **1** with those of known related bromotyrosine alkaloids, such as psammaplysins A (**4**) [12,13,14] and F (**3**), suggested that **1** possessed an 8,10-dibromo-9-methoxy-1,6-dioxa-2-azaspiro[4.6]undeca-2,7,9-trien-4-ol unit (C-1~C-8, 1-O, 2-Br, 3-OCH_3_, 4-Br, 6-O, 7-OH, and 8-N) and two 11-*N*-methylmoloka’iamine [15] units (C-10~C-21, 9-N, 12-O, 14-Br, 18-Br, and 20-N; C-23~C-34, 22-N, 25-O, 27-Br, 31-Br, and 33-N). The correlations observed in the ^1^H-^1^H COSY and HMBC spectra of **1** supported the presumption, see Figure 1, Figures S6 and S8. The HMBC correlations between *N*-methylene protons H_2_-20 (δ_H_ 3.52) and a carbonyl carbon C-22 (δ_C_ 161.2), *N*-methyl protons H_3_-21 (δ_H_ 2.88) and C-22, and *N*-methylene protons H_2_-23 (δ_H_ 3.45) and C-22 revealed that 20-N and 22-N were connected through a carbonyl group at C-22, see Figure 2. Though no correlation across the C-8-C-9 bond was observed in the HMBC spectrum of **1**, the HMBC correlation between *N*-methylene protons H_2_-10 (δ_H_ 3.65) and a carbonyl carbon C-9 (δ_C_ 161.5) and the molecular formula of **1** implied that C-8 and 9-N were connected through a carbonyl group at C-9, as shown in Figure 1. Thus, the gross structure of **1** was fully elucidated. The absolute configurations at C-6 and C-7 of **1** were assigned as *R* and *R*, respectively, since the NMR data of the 8,10-dibromo-9-methoxy-1,6-dioxa-2-azaspiro[4.6]undeca-2,7,9-trien-4-ol moiety of **1** and the pattern of the ECD spectrum of **1**, see Figure S9, were coincident with those of psammaplysin A (**4**), a known related bromotyrosine alkaloid whose absolute configuration was established.

**Figure 3:**
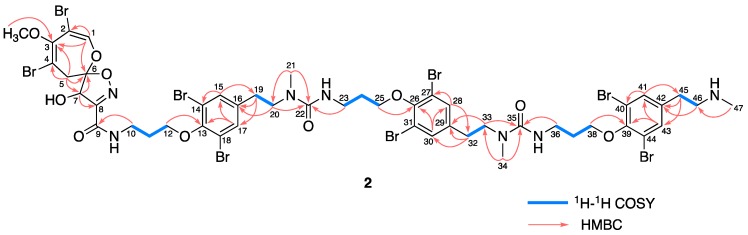
Selected 2D NMR correlations for ceratinadin F (**2**).

Ceratinadin F (**2**) was obtained as an optically active colorless amorphous solid. The molecular formula of **2** was established as C_48_H_57_Br_8_N_7_O_10_ by HRESIMS data, see Figures S10, S11, and S12. The spectroscopic data (UV, IR, and NMR spectra) of **2** were analogous to those of ceratinadin E (**1**) except for the integral values of ^1^H NMR signals ascribed to 11-*N*-mehtylmoloka’iamine units were 1.5 times as large as that of **1**, see Figures S13, S14, S15, S16, and S17. These data suggested that **2** was an analog of **1** possessing an additional 11-*N*-methylmoloka’iamine unit at the terminal amino group of **1**. The presumption was supported by the correlations observed in the ^1^H-^1^H COSY and HMBC spectra of **2**, see Figure 3. Since the NMR data of the 8,10-dibromo-9-methoxy-1,6-dioxa-2-azaspiro[4.6]undeca-2,7,9-trien-4-ol moiety of **2** and the pattern of the ECD spectrum of **2**, see Figure S18, were coincident with those of psammaplysin A (**4**), the absolute configurations at C-6 and C-7 of **2** were assigned as *R* and *R*, respectively.

The antimalarial activities of psammaplysin F (**3**), ceratinadin E (**1**), and ceratinadin F (**2**) against a drug-resistant and a drug-sensitive strains of *Plasmodium falciparum* (K1 and FCR3 strains, respectively) were examined, see Table 2 [16]. Though the antimalarial activities of psammaplysin F (**3**) against a drug-resistant and a drug-sensitive strains of *P. falciparum* (Dd2 and 3D7 strains, respectively) were reported [3,4], **3** also exhibited antimalarial activities against K1 (IC_50_ 3.77 μg/mL) and FCR3 (IC_50_ 2.45 μg/mL) strains in vitro. Ceratinadin E (**1**) exhibited slightly greater antimalarial activities against both K1 (IC_50_ 1.03 μg/mL) and FCR3 (IC_50_ 0.77 μg/mL) strains in vitro with better selectivity indexes than **3**, while ceratinadin F (**2**) did not show significant antimalarial activity against the K1 strain (IC_50_ >12.5 μg/mL).

## 3. Discussion

About five hundred bromotyrosine alkaloids have been isolated from marine Verongid sponges [2 and references therein]. Among them, thirty seven bromotyrosine alkaloids possessing the 1,6-dioxa-2-azaspiro[4.6]undecane skeleton have been reported from marine sponges belonging to the genus *Aplysinella*, *Hyattella*, *Psedoceratina*, and *Suberea*. The biosynthetic pathway of 3,5-dibromo-l-tyrosine from l-phenylalanine and l-tyrosine by bromoperoxidase was proposed by Rinehert et al. [17]. Subsequently, the biosynthetic pathway of the 1,6-dioxa-2-azaspiro[4.6]undecane skeleton from 3,5-dibromo-l-tyrosine was proposed by Scheuer and Clardy et al. [13]. The antimalarial activity of eleven bromotyrosine alkaloids possessing the 1,6-dioxa-2-azaspiro[4.6]undecane skeleton have been examined so far and psammaplysins F (**3**) and H and 19-hydroxypsammaplysin E were known to exhibit the significant activity [4,5,6]. Though psammaplysin F (**3**) displayed the most potent activity against a drug-resistant strain of *P. falciparum* (Dd2 strain) among those compounds, ceratinadin E (**1**) showed the greater antimalarial activity against a drug-resistant strain of *P. falciparum* (K1 strain) in vitro with a better selectivity index than **3**. An additional 11-*N*-methylmoloka’iamine unit might enhanced the activity, however ceratinadin F (**2**) did not show significant antimalarial activity. As Garson et al. reported that excessive lipophilicity decreases antimalarial activity [6], the lipophilicity of **2** might be too high. Though the antimalarial activity against drug-resistant strains of *P. falciparum* was unknown, psammaplysin H, a derivative of **3** terminating in a trimethylaminium group instead of a methylamino group, exhibited the greater antimalarial activity against a drug-sensitive strain of *P. falciparum* than **3** without significant cytotoxicity. The antimalarial activity of the derivative of **1** terminating in a trimethylaminium group, which might be isolated from sponges or derived from **1**, is quite fascinating.

## 4. Materials and Methods 

### 4.1. General Experimental Procedures

The optical rotations were recorded on a JASCO P-2200 polarimeter. The UV spectra were recorded on a JASCO Ubest-55 spectrophotometer. The IR spectra were recorded on a JASCO FT/IR-420 spectrophotometer. The ECD spectra were recorded on a JASCO J-1500 spectropolarimeter. ^1^H and ^13^C NMR spectra were recorded on a Bruker Avance II 600 MHz NMR spectrometer equipped with a cryoplatform using 3.0 mm micro cells (Shigemi Co., Ltd., Tokyo, Japan) for CD_3_OD. The 3.35 ppm resonance of residual CD_2_HOD in CD_3_OD was used as the internal references for ^1^H NMR spectra. The 49.8 ppm resonances of CD_3_OD were used as the internal reference for the ^13^C NMR spectra. The MS spectra were recorded on a JEOL JMS-T100LP spectrometer. The flash column chromatography was performed with a Biotage Isolera flash purification system.

### 4.2. Extraction and Isolation

The sponge *Pseudoceratina* sp. (order Verongida; family Aplysinellidae) was collected at Okinawa, Japan. The sponge was kept frozen until used. The sponge (0.4 kg, wet weight) was extracted with MeOH (500 mL × 3) and the extract was concentrated in vacuo. The residue (38.06 g) was partitioned between EtOAc (300 mL × 3) and H_2_O (300 mL) to afford the EtOAc-soluble material (2.45 g). A part of the EtOAc-soluble material (1.00 g) was fractionated by silica gel column chromatography [Silica gel 60N (spherical, neutral, 40–50 µm), Kanto Chemical Co., Inc.; 38 × 320 mm; eluent CHCl_3_/MeOH, 100:0 to 0:100] to give 12 fractions (Fr.1~12). A part (100.0 mg) of the fraction Fr.11 (257.6 mg) was passed through a short C_18_ column (Sep-Pak C18 Plus Short Cartridge, Waters; eluent MeOH, 100:0), and the eluted material (90.4 mg) was separated by C_18_ flash column chromatography (Isolera SNAP Ultra C18 12 g, Biotage; eluent MeOH/H_2_O/TFA, 10:90:0 to 100:0:0.1) and C_18_ HPLC (COSMOSIL 5C_18_-AR-II, 10 × 250 mm, Nacalai Tesque Inc.; eluent MeOH/H_2_O/TFA, 60:40:0.1; flow rate 2.5 mL/min; UV detection at 254 nm) to yield psammaplysin F (**3**, *t*_R_ 6.1 min, 10.8 mg, 0.017% wet weight), ceratinadin E (**1**, *t*_R_ 8.8 min, 4.6 mg, 0.0073% wet weight), and ceratinadin F (**2**, *t*_R_ 15.6 min, 0.4 mg, 0.00063% wet weight). 

### 4.3. Ceratinadin E (***1***)

Colorless amorphous solid; [α]${}_{D}^{24}$ −45.1 (*c* = 0.48, MeOH); UV (MeOH) λ_max_ 206 (ε 117744), 263 (ε 10233) nm; IR (film/KBr) ν_max_ 3338, 2935, 2880, 1671, 1624, 1541, 1257, 1200, 1133 cm^−1^; ECD (MeOH) λ_max_ (Δε) 212 (−17.44), 242 (+7.73), 288 (−1.14) nm; ^1^H NMR and ^13^C NMR data, as shown in Table 1; ESIMS *m/z* 1133, 1135, 1137, 1139, 1141, 1143, 1145 (1:6:15:20:15:6:1, [M + H]^+^); HRESIMS *m/z* 1139.80628 [M + H]^+^ (calcd for C_35_H_42_^79^Br_3_^81^Br_3_N_5_O_8_, 1139.80722).

### 4.4. Ceratinadin F (***2***)

Colorless amorphous solid; [α]${}_{D}^{24}$ −25.0 (*c* = 0.18, MeOH); UV (MeOH) λ_max_ 206 (ε 60692), 262 (ε 4308) nm; IR (film/KBr) ν_max_ 3413, 2926, 2849, 1676, 1581, 1210, 1183, 1140 cm^−1^; ECD (MeOH) λ_max_ (Δε) 209 (−11.32), 243 (+4.34), 278 (−0.18) nm; ^1^H NMR and ^13^C NMR data, as shown in Table 1; ESIMS *m/z* 1523, 1525, 1527, 1529, 1531, 1533, 1535, 1537, 1539 (1:8:28:56:70:56:28:8:1, [M + H]^+^); HRESIMS *m/z* 1531.76647 [M + H]^+^ (calcd for C_48_H_58_^79^Br_4_^81^Br_4_N_7_O_10_, 1531.76303).

## 5. Conclusions

In conclusion, two new bromotyrosine alkaloids, ceratinadins E (**1**) and F (**2**), were isolated from an Okinawan marine sponge *Pseudoceratina* sp. as well as a known bromotyrosine alkaloid, psammaplysin F (**3**). Ceratinadins E (**1**) and F (**2**) are new bromotyrosine alkaloids possessing an 8,10-dibromo-9-methoxy-1,6-dioxa-2-azaspiro[4.6]undeca-2,7,9-trien-4-ol unit with two or three 11-*N*-methylmoloka’iamine units connected by carbonyl groups, respectively. Ceratinadin E (**1**) and psammaplysin F (**3**) exhibited antimalarial activities against a drug-resistant and a drug-sensitive strains of *Plasmodium falciparum* (K1 and FCR3 strains, respectively) in vitro, while ceratinadin F (**2**) did not show significant antimalarial activity. Further isolation, structure elucidation, and structure-activity relationship studies of this type of alkaloids are required for the development of new antimalarial drugs.

## Figures and Tables

**Table 1:** ^1^H and ^13^C NMR data of ceratinadins E (**1**) and F (**2**) in CD_3_OD.

Ceratinadin E (1)					Ceratinadin F (2)				
Position	δ_H_ *^a^*	Multi (*J* in Hz)	δ_C_ *^b^*	Multi	Position	δ_H_ *^a^*	Multi (*J* in Hz)	δ_C_ *^b^*	Multi
1	7.18	s	147.6	d	1	7.17	s	147.6	d
2	−		105.1	s	2	−		105.1	s
3	−		150.7	s	3	−		150.7	s
3-OCH_3_	3.69 *^d^*	s	60.1	q	3-OCH_3_	3.69 *^d^*	s	60.1	q
4	-		105.3	s	4	-		105.3	s
5a	3.40	m*^e^*	39.1	t	5a	3.41	d (16.4)	39.1	t
5b	3.11	d (15.7)			5b	3.10	d (16.4)		
6	-		121.7	s	6	-		121.6	s
7	5.03	s	81.2	d	7	5.02	s	81.2	d
8	-		159.5	s	8	-		159.5	s
9	-		161.5	s	9	-		161.5	s
10	3.65 *^c^*	td (6.6, 1.2)	38.8	t	10	3.65 *^c^*	td (6.6, 2.7)	38.8	t
11	2.15 *^c^*	dt (6.6, 6.6)	31.3	t	11	2.15 *^c^*	dt (6.6, 6.6)	31.3	t
12	4.08 *^c^*	m*^e^*	72.9	t	12	4.08 *^c^*	m*^e^*	72.9	t
13	-		153.5	s	13	-		153.5	s
14, 18	-		119.7	s	14, 18	-		119.8	s
15, 17	7.49	s	135.2	d	15, 17	7.49	s	135.2	d
16	-		140.8	s	16	-		140.8	s
19	2.81 *^c^*	t (7.1)	34.6	t	19	2.81 *^c^*	m*^e^*	34.8	t
20	3.52 *^c^*	t (7.1)	51.7	t	20	3.51 *^c^*	m*^e^*	51.7	t
21	2.88 *^d^*	s	35.8	q	21	2.88 *^d^*	s	35.8	q
22	-		161.2	s	22	-		161.2	s
23	3.45 *^c^*	m*^e^*	40.1	t	23	3.45 *^c^*	m*^e^*	40.2	t
24	2.06 *^c^*	dt (6.4, 6.4)	32.4	t	24	2.05 *^c^*	m*^e^*	32.5	t
25	4.09 *^c^*	m*^e^*	73.7	t	25	4.08 *^c^*	m*^e^*	73.7	t
26	-		154.4	s	26	-		153.6	s
27, 31	-		120.3	s	27, 31	-		119.8	s
28, 30	7.59	s	135.2	d	28, 30	7.49	s	135.2	d
29	-		138.0	s	29	-		140.7	s
32	2.98	br t (7.6)	33.0	t	32	2.81	m*^e^*	34.8	t
33	3.27	br t (7.6)	51.9	t	33	3.52	m*^e^*	51.7	t
34	2.70 *^d^*	s	34.7	q	34	2.88 *^d^*	s	35.8	q
					35	-		161.2	s
					36	3.44 *^c^*	m*^e^*	40.2	t
					37	2.05 *^c^*	m*^e^*	32.4	t
					38	4.05 *^c^*	m*^e^*	73.6	t
					39	-		154.5	s
					40, 44	-		120.3	s
					41, 43	7.56	s	135.2	d
					42	-		138.0	s
					45	2.93	br t (7.8)	33.0	t
					46	3.18	m*^e^*	51.9	t
					47	2.70 *^d^*	s	34.7	q

*^a^* 600 MHz. *^b^* 150 MHz. *^c^* 2H. *^d^* 3H. *^e^ J*-values were not determined because of overlapping with other signals.

**Table 2:** Antimalarial activities of psammaplysin F (**3**), ceratinadin E (**1**), and ceratinadin F (**2**).

	Antimalarial Activity *^a^*		Cytotoxicity *^a^*	Selectivity Index	
	K1 *^b^*	FCR3 *^c^*	MRC-5*^d^*	MRC-5/K1	MRC-5/FCR3
Psammaplysin F (**3**)	3.77	2.45	12.65	3.4	5.2
Ceratinadin E (**1**)	1.03	0.77	15.99	15.5	20.8
Ceratinadin F (**2**)	>12.5	-*^e^*	>50	>4	-*^e^*
Chloroquine *^f^*	0.34	0.035	>25.80	>75.9	>737.1
Artemisinin *^f^*	0.010	0.0088	>14.12	>1412	>1604.5

*^a^* IC_50_ (μg/mL). *^b^* Drug-resistant *P. falciparum* strain. *^c^* Drug-sensitive *P. falciparum* strain. *^d^* Normal human embryonic lung fibroblast. *^e^* not examined. *^f^* Existing antimalarial drug.

## Supplementary Materials

The following are available online at http://www.mdpi.com/1660-3397/16/12/463/s1, Figure S1: ESIMS spectrum (positive ion mode) of ceratinadin E (**1**), Figure S2: Expanded ESIMS spectrum (positive ion mode) of ceratinadin E (**1**), Figure S3: HRESIMS data (positive ion mode) of ceratinadin E (**1**), Figure S4: ^1^H NMR spectrum of ceratinadin E (**1**) in CD_3_OD (600 MHz), Figure S5: ^13^C NMR spectrum of ceratinadin E (**1**) in CD_3_OD (150 MHz), Figure S6: ^1^H-^1^H COSY spectrum of ceratinadin E (**1**) in CD_3_OD (600 MHz), Figure S7: HSQC spectrum of ceratinadin E (**1**) in CD_3_OD (600 MHz), Figure S8: HMBC spectrum of ceratinadin E (**1**) in CD_3_OD (600 MHz), Figure S9: ECD spectrum of ceratinadin E (**1**) in CH_3_OH, Figure S10: ESIMS spectrum (positive ion mode) of ceratinadin F (**2**), Figure S11: Expanded ESIMS spectrum (positive ion mode) of ceratinadin F (**2**), Figure S12: HRESIMS data (positive ion mode) of ceratinadin F (**2**), Figure S13: ^1^H NMR spectrum of ceratinadin F (**2**) in CD_3_OD (600 MHz), Figure S14: ^13^C NMR spectrum of ceratinadin F (**2**) in CD_3_OD (150 MHz), Figure S15: ^1^H-^1^H COSY spectrum of ceratinadin F (**2**) in CD_3_OD (600 MHz), Figure S16: HSQC spectrum of ceratinadin F (**2**) in CD_3_OD (600 MHz), Figure S17: HMBC spectrum of ceratinadin F (**2**) in CD_3_OD (600 MHz), Figure S18: ECD spectrum of ceratinadin F (**2**) in CH_3_OH.
