# Supplementary material for: Ceratinadins E and F, New Bromotyrosine Alkaloids from an Okinawan Marine Sponge Pseudoceratina sp
Source: Mar Drugs. 2018 Nov 23;16(12):463. doi: 10.3390/md16120463 (PMC6316200; doi:10.3390/md16120463)

Supplementary Materials

Ceratinadins E and F, new bromotyrosine alkaloids from an Okinawan marine sponge *Pseudoceratina* sp.

*Shin-ichiro Kurimoto[1], Taito Ohno[1], Rei Hokari[2], Aki Ishiyama[2], Masato Iwatsuki[2], Satoshi Ōmura[2], Jun'ichi Kobayashi[3] and Takaaki Kubota[1,]**

[1]Showa Pharmaceutical University, 3-3165 Higashi-Tamagawagakuen, Machida, Tokyo 194-8543, Japan.

[2]Kitasato Institute for Life Sciences, Kitasato University, 5-9-1 Shirokane, Minato-ku, Tokyo 108-8641, Japan.

[3]Graduate School of Pharmaceutical Sciences, Hokkaido University, Sapporo 060-0812, Japan.

Table of Contents:

Figure S1. ESIMS spectrum (positive ion mode) of ceratinadin E (**1**).
Figure S2. Expanded ESIMS spectrum (positive ion mode) of ceratinadin E (**1**).
Figure S3. HRESIMS data (positive ion mode) of ceratinadin E (**1**).
Figure S4. ^{1}H NMR spectrum of ceratinadin E (**1**) in CD_3OD (600 MHz).
Figure S5. ^{13}C NMR spectrum of ceratinadin E (**1**) in CD_3OD (150 MHz).
Figure S6. ^{1}H-^{1}H COSY spectrum of ceratinadin E (**1**) in CD_3OD (600 MHz).
Figure S7. HSQC spectrum of ceratinadin E (**1**) in CD_3OD (600 MHz).
Figure S8. HMBC spectrum of ceratinadin E (**1**) in CD_3OD (600 MHz).
Figure S9. ECD spectrum of ceratinadin E (**1**) in CH_3OH.
Figure S10. ESIMS spectrum (positive ion mode) of ceratinadin F (**2**).
Figure S11. Expanded ESIMS spectrum (positive ion mode) of ceratinadin F (**2**).
Figure S12. HRESIMS data (positive ion mode) of ceratinadin F (**2**).
Figure S13. ^{1}H NMR spectrum of ceratinadin F (**2**) in CD_3OD (600 MHz).
Figure S14. ^{13}C NMR spectrum of ceratinadin F (**2**) in CD_3OD (150 MHz).
Figure S15. ^{1}H-^{1}H COSY spectrum of ceratinadin F (**2**) in CD_3OD (600 MHz).
Figure S16. HSQC spectrum of ceratinadin F (**2**) in CD_3OD (600 MHz).
Figure S17. HMBC spectrum of ceratinadin F (**2**) in CD_3OD (600 MHz).
Figure S18. ECD spectrum of ceratinadin F (**2**) in CH_3OH.

Figure S1. ESIMS spectrum (positive ion mode) of ceratinadin E (**1**).

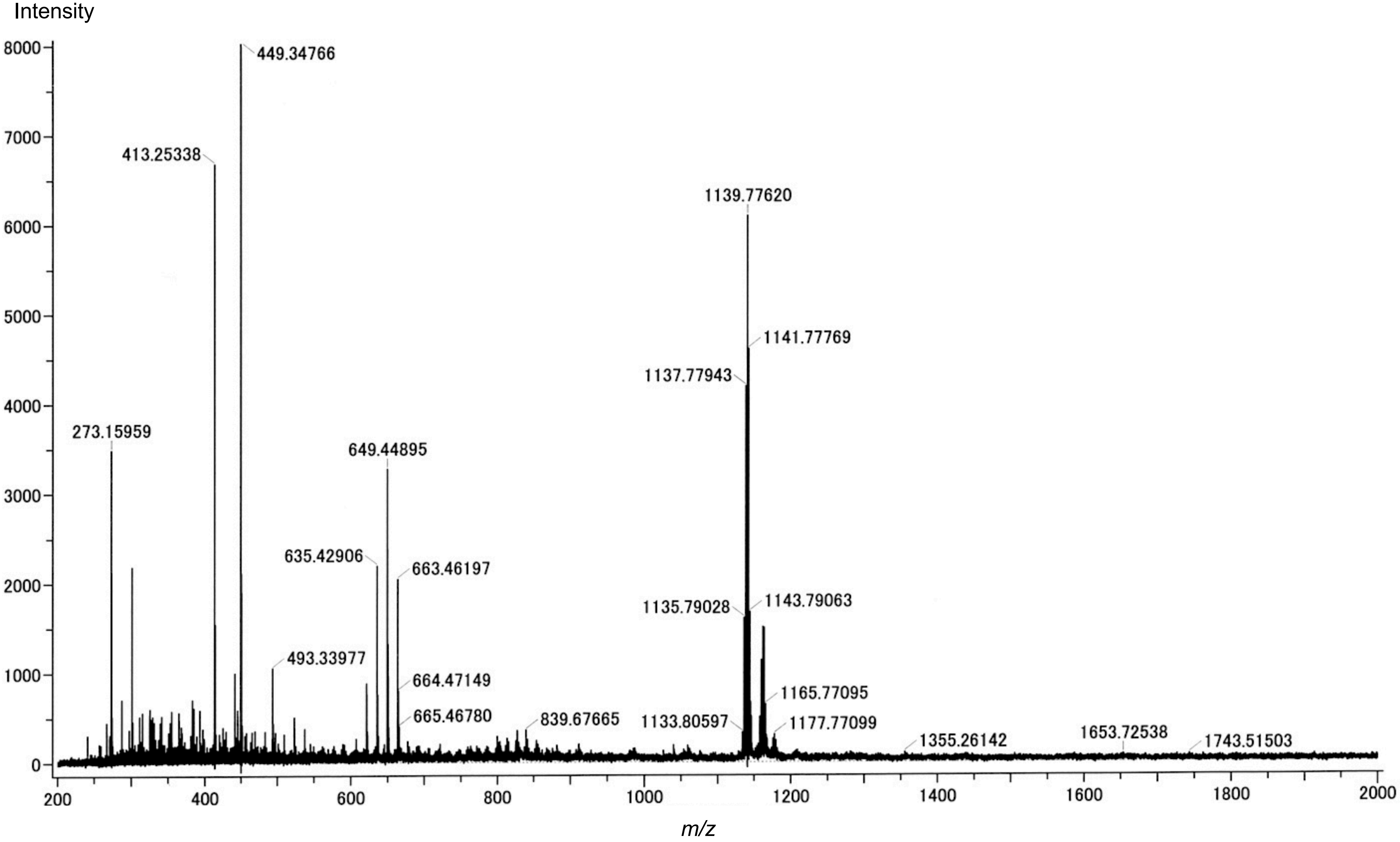

Figure S2. Expanded ESIMS spectrum (positive ion mode) of ceratinadin E (**1**).

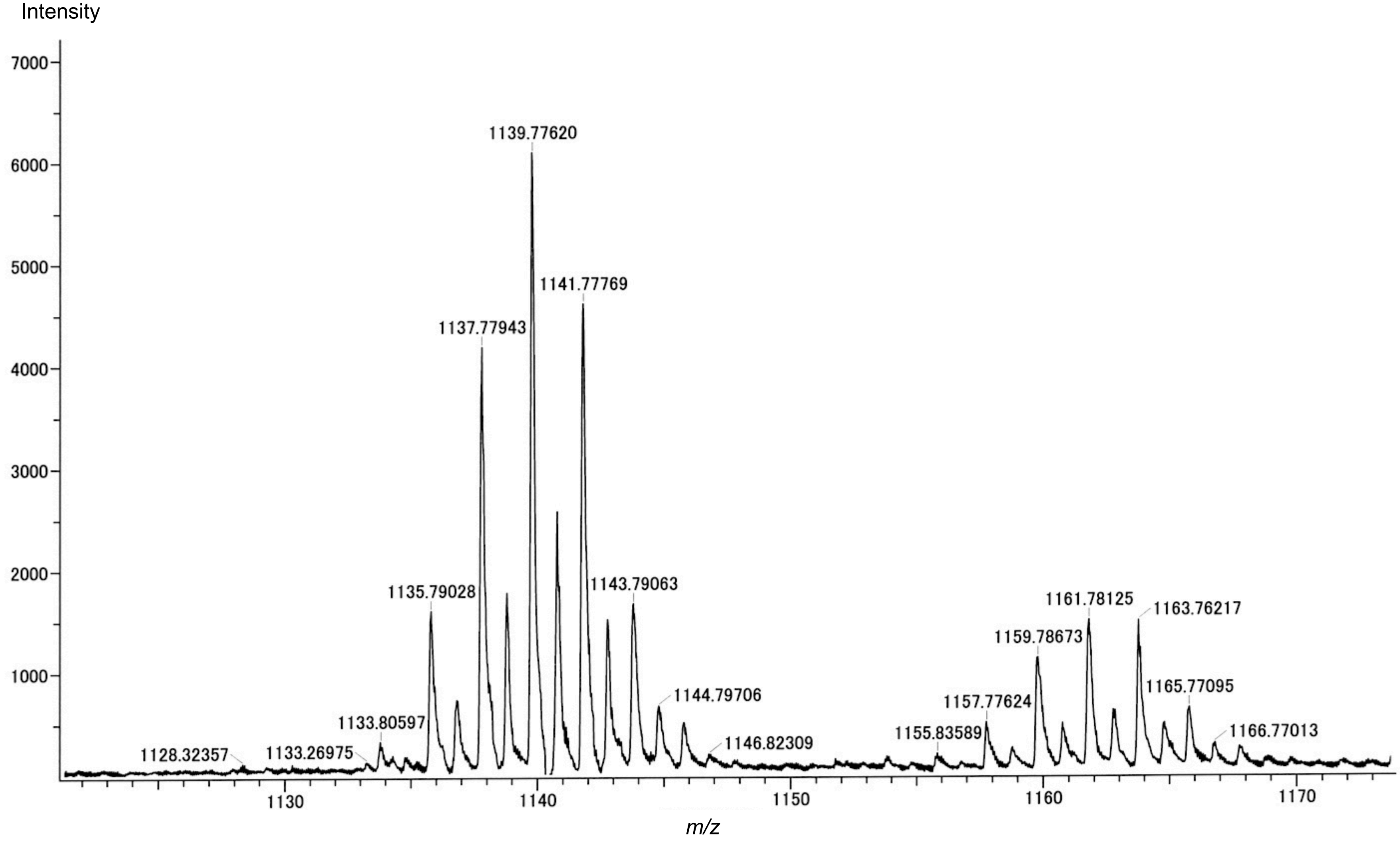

Figure S3. HRESIMS (positive) data of ceratinadin E (**1**).

m/z	Intensity	Theo. Mass	Delta (mmu)	Composition	RDB equiv.
1139.80628	184575.00	1139.80722	−0.94	$^{12}C_{35}{}^{1}H_{42}{}^{79}Br_{3}{}^{81}Br_{3}{}^{14}N_{5}{}^{16}O_{8}$	14.5

Figure S4. ^{1}H NMR spectrum of ceratinadin E (**1**) in CD_3OD (600 MHz).

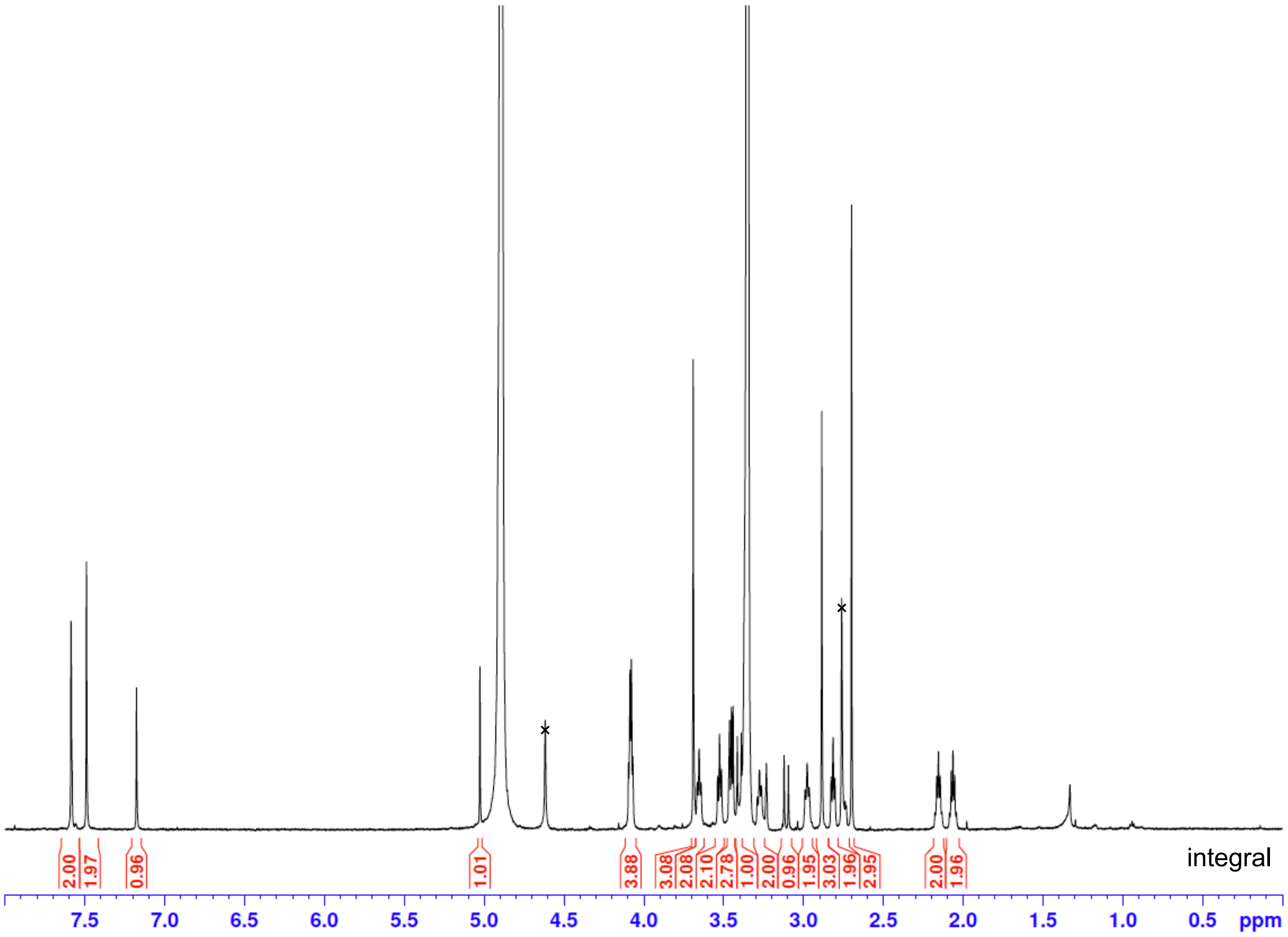

Figure S5. ^{13}C NMR spectrum of ceratinadin E (**1**) in CD_3OD (150 MHz).

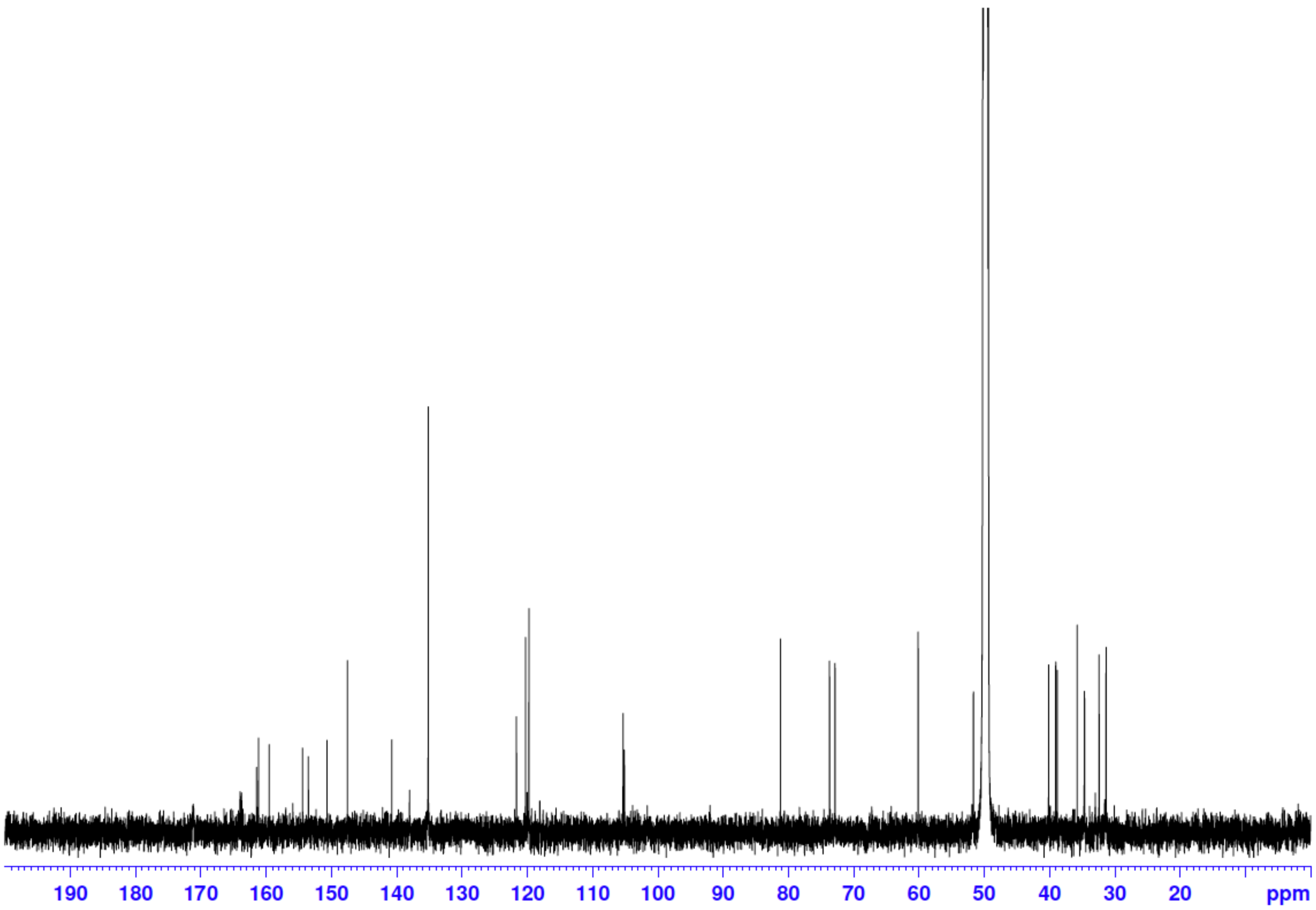

Figure S6. ^{1}H-^{1}H COSY spectrum of ceratinadin E (**1**) in CD_3OD (600 MHz).

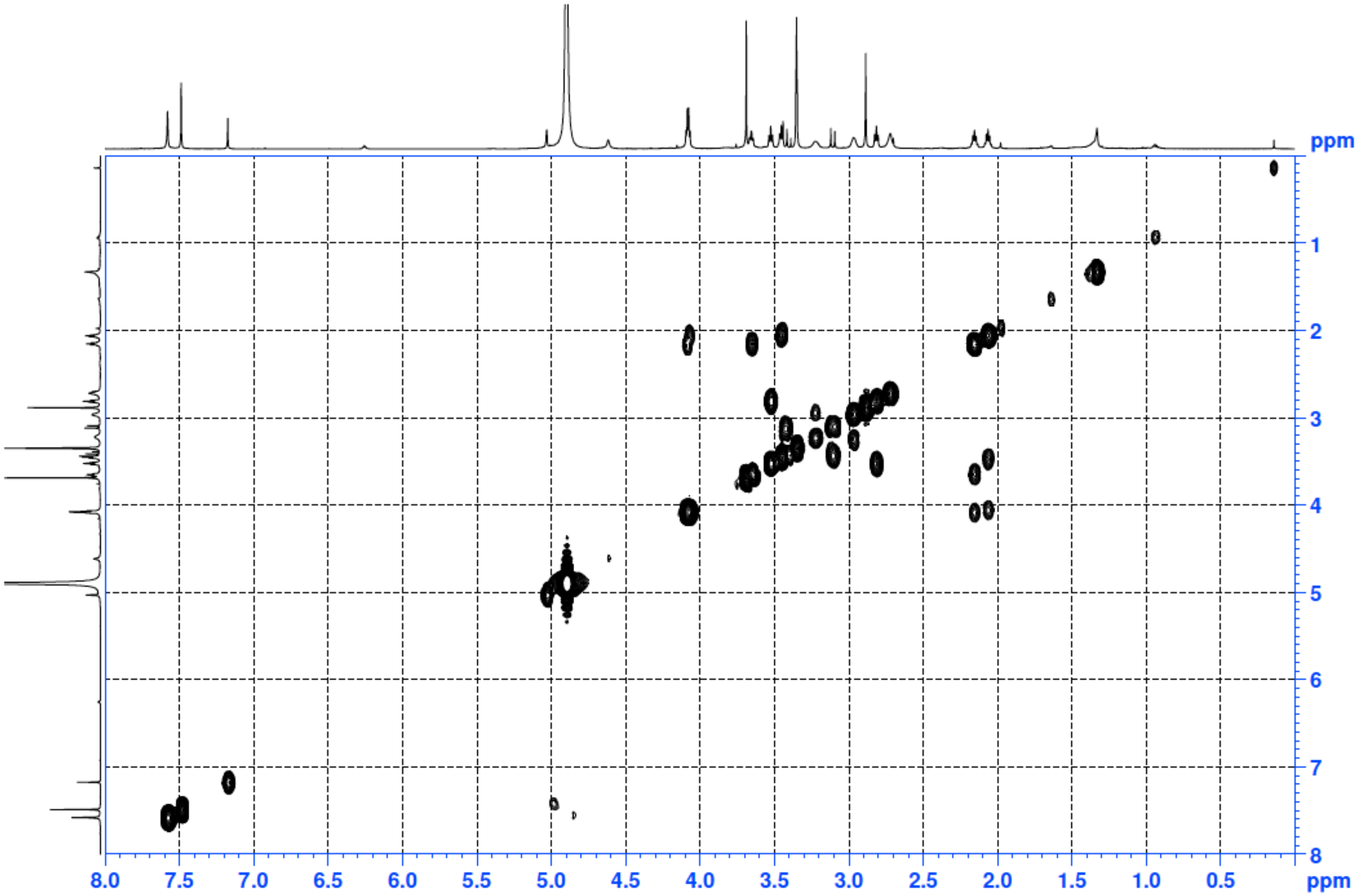

Figure S7. HSQC spectrum of ceratinadin E (**1**) in CD_3OD (600 MHz).

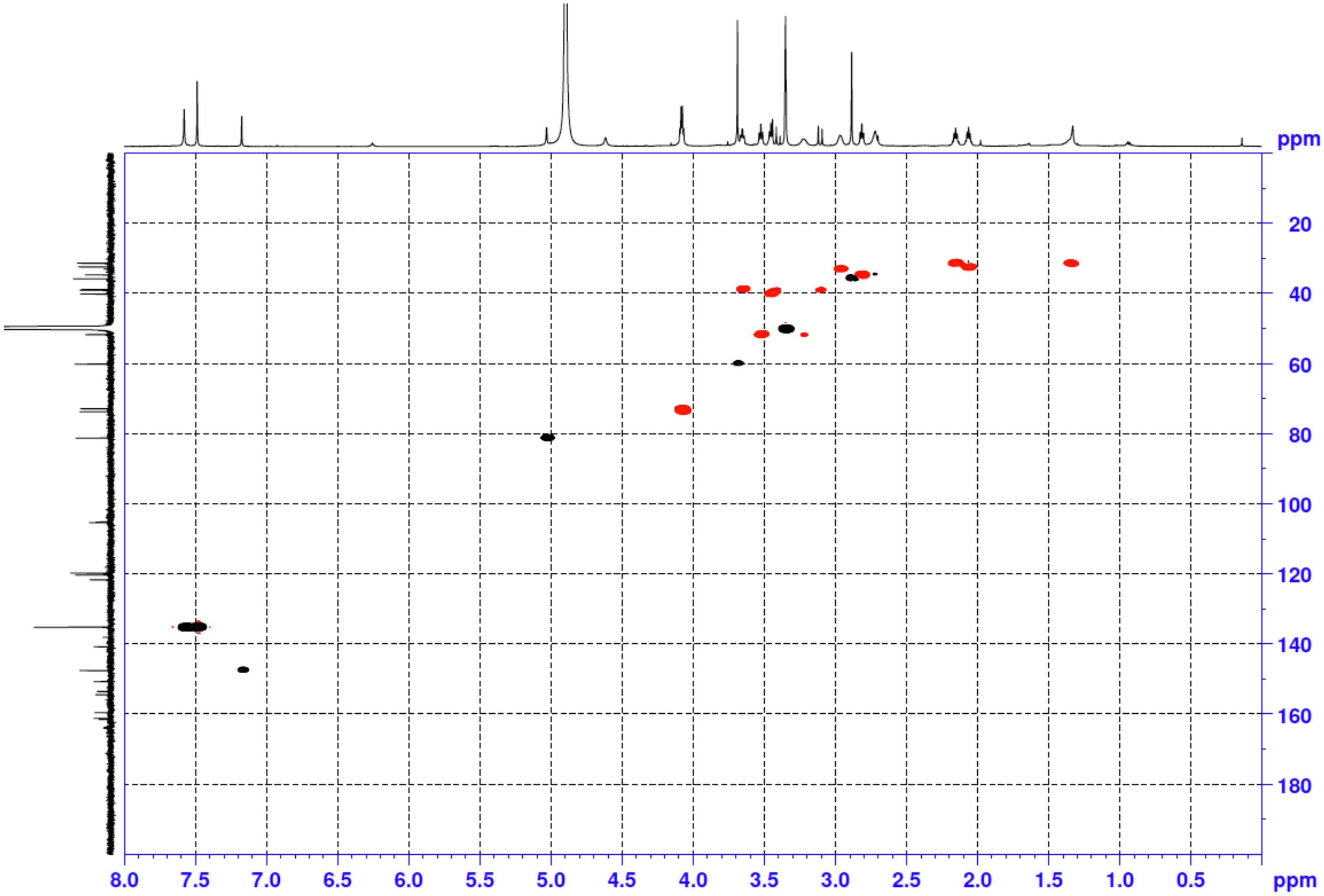

Figure S8. HMBC spectrum of ceratinadin E (**1**) in CD_3OD (600 MHz).

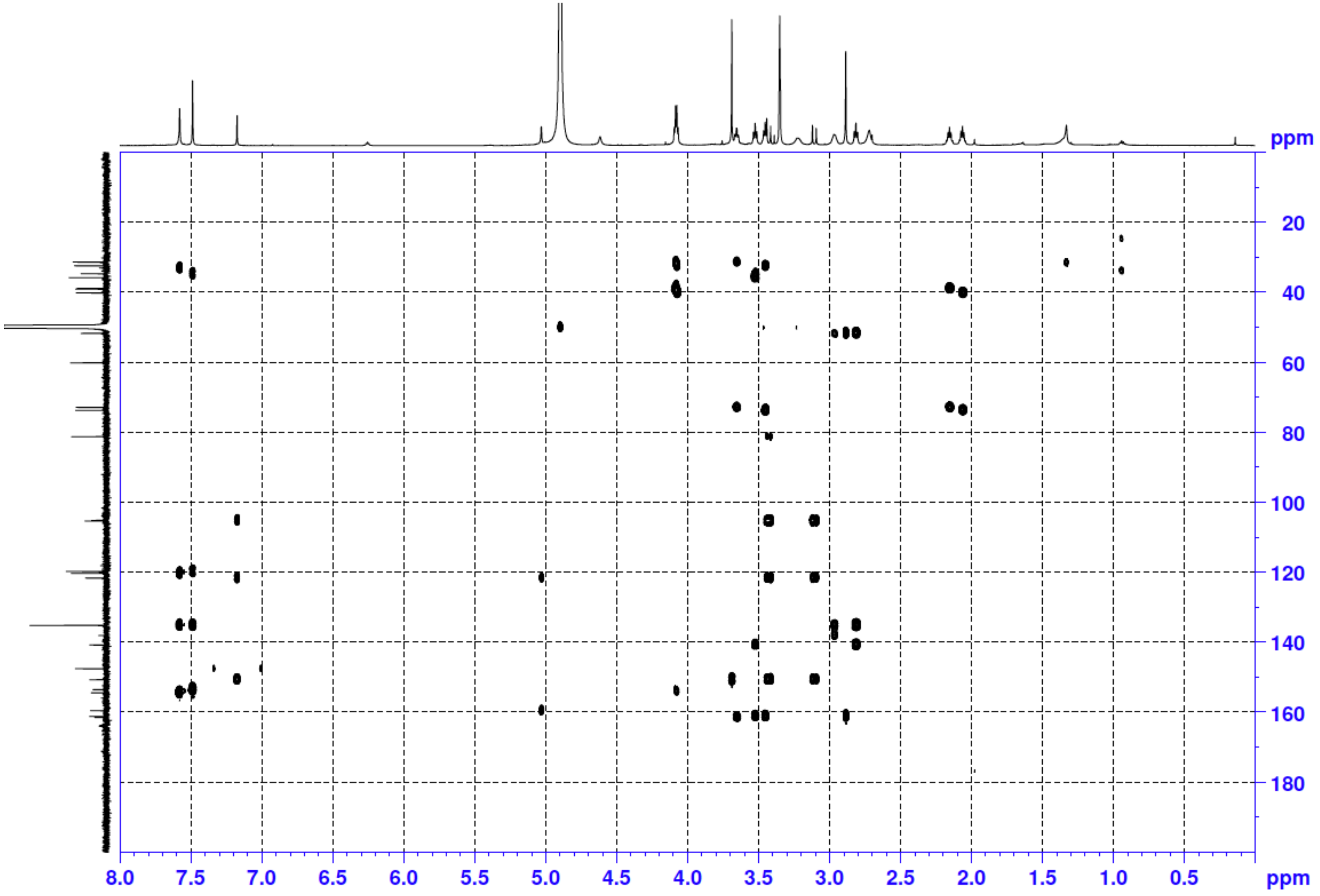

Figure S9. ECD spectrum of ceratinadin E (**1**) in CH_3OH.

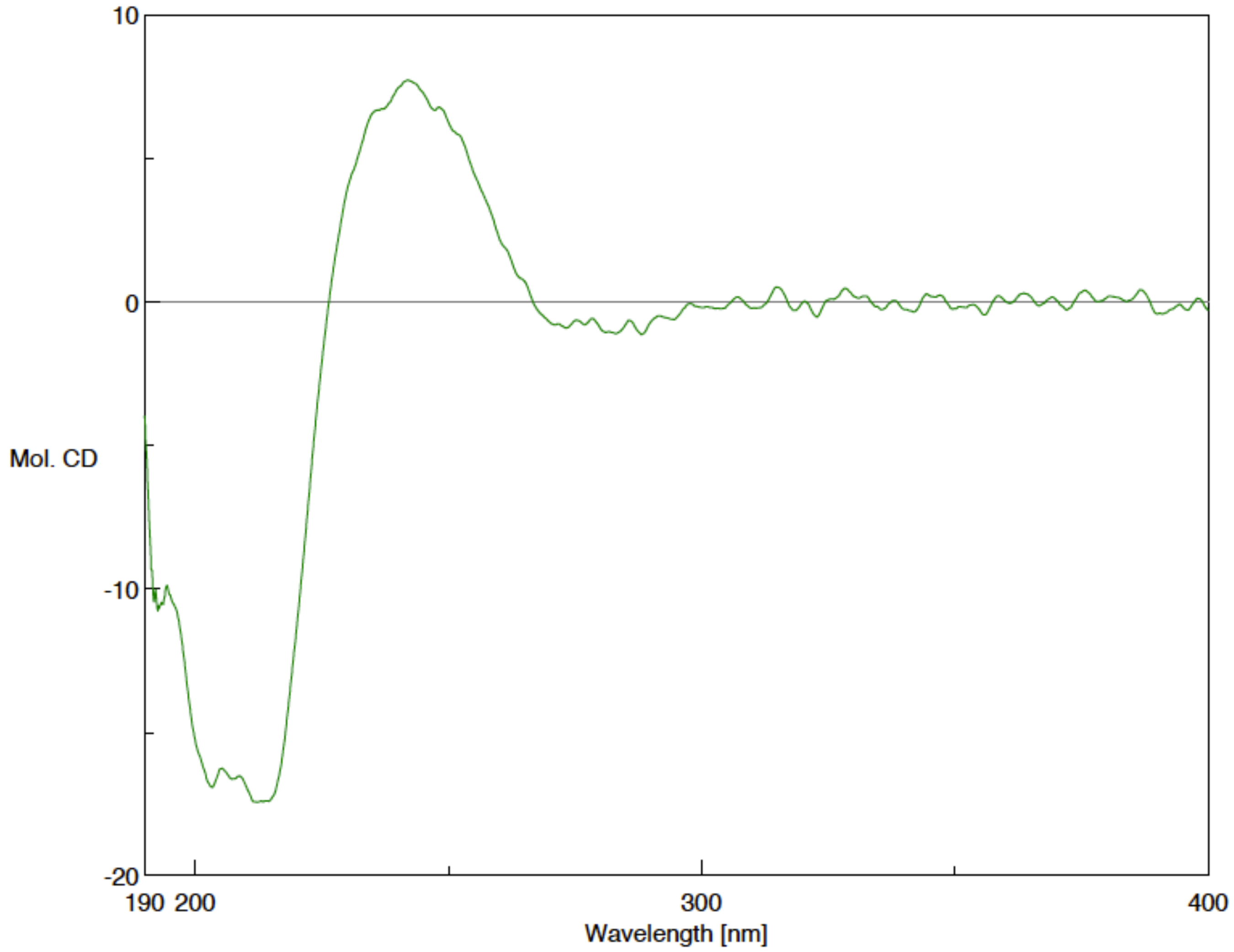

Figure S10. ESIMS spectrum (positive ion mode) of ceratinadin F (**2**).

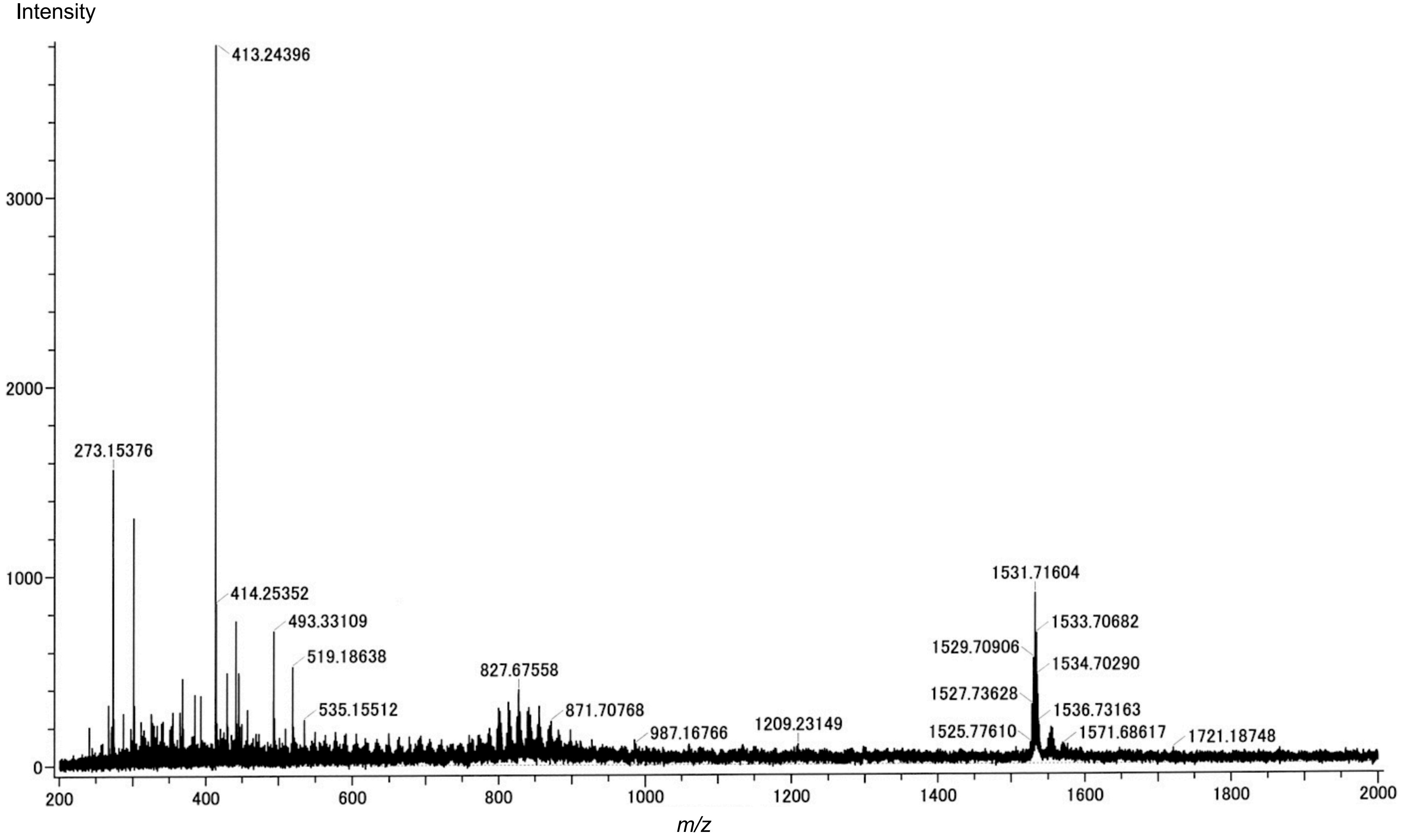

Figure S11. Expanded ESIMS spectrum (positive ion mode) of ceratinadin F (**2**).

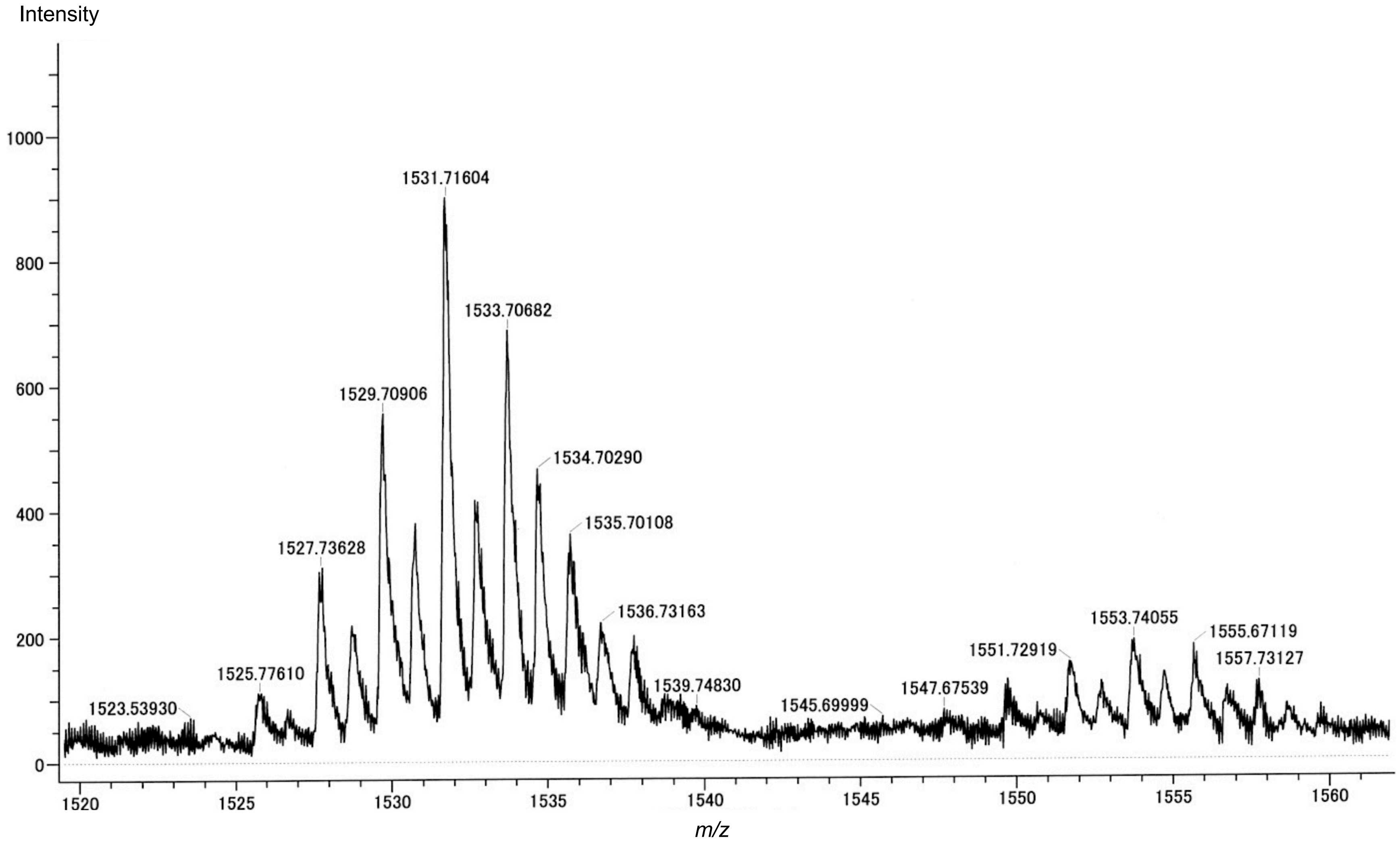

Figure S12. HRESIMS (positive) data of ceratinadin F (**2**).

m/z	Intensity	Theo. Mass	Delta (mmu)	Composition	RDB equiv.
1531.76647	508623.19	1531.76303	3.43	$^{12}C_{48}{}^{1}H_{58}{}^{79}Br_{4}{}^{81}Br_{4}{}^{14}N_{7}{}^{16}O_{10}$	19.5

Figure S13. ^{1}H NMR spectrum of ceratinadin F (**2**) in CD_3OD (600 MHz).

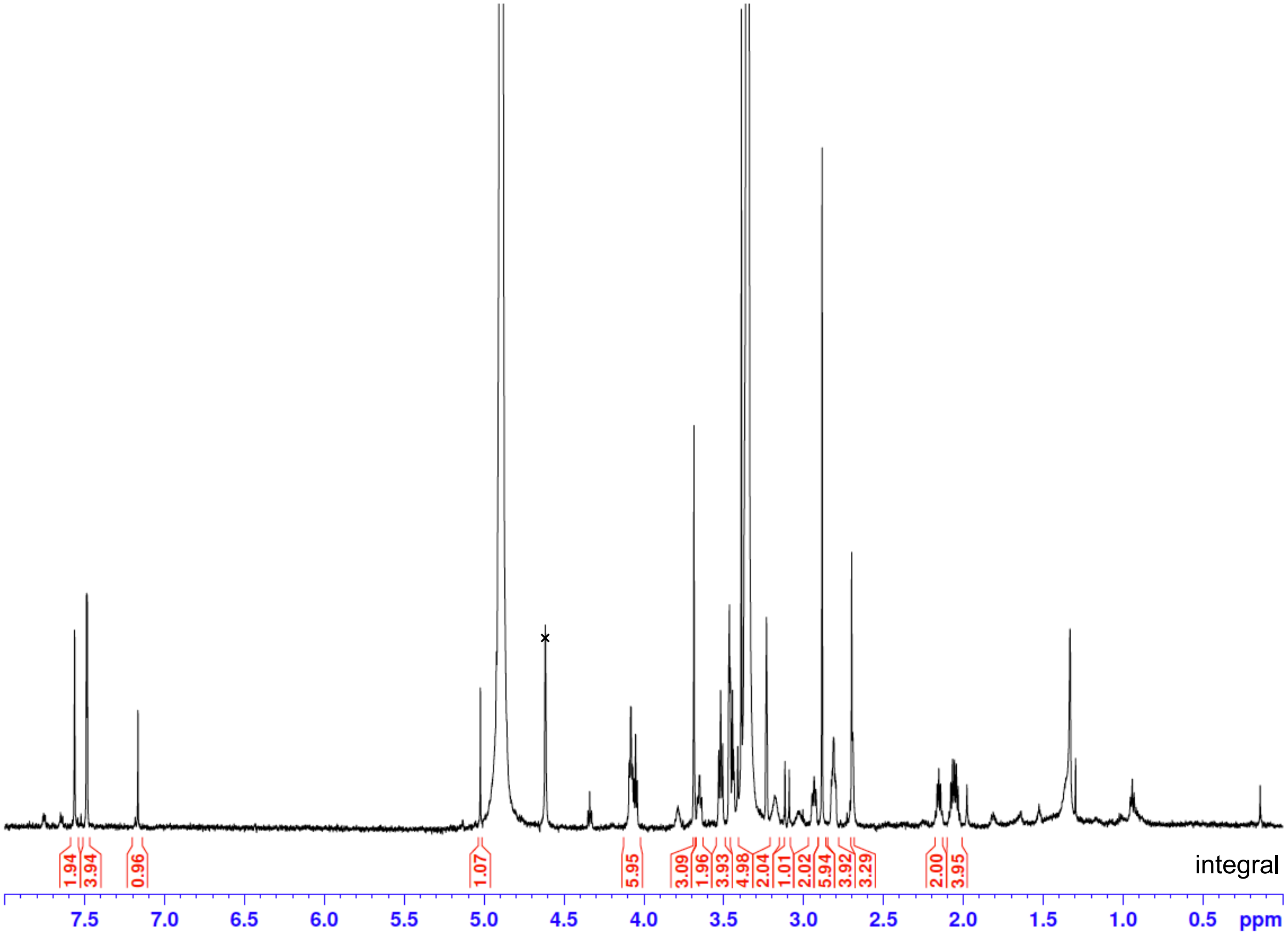

Figure S14. ^{13}C NMR spectrum of ceratinadin F (**2**) in CD_3OD (150 MHz).

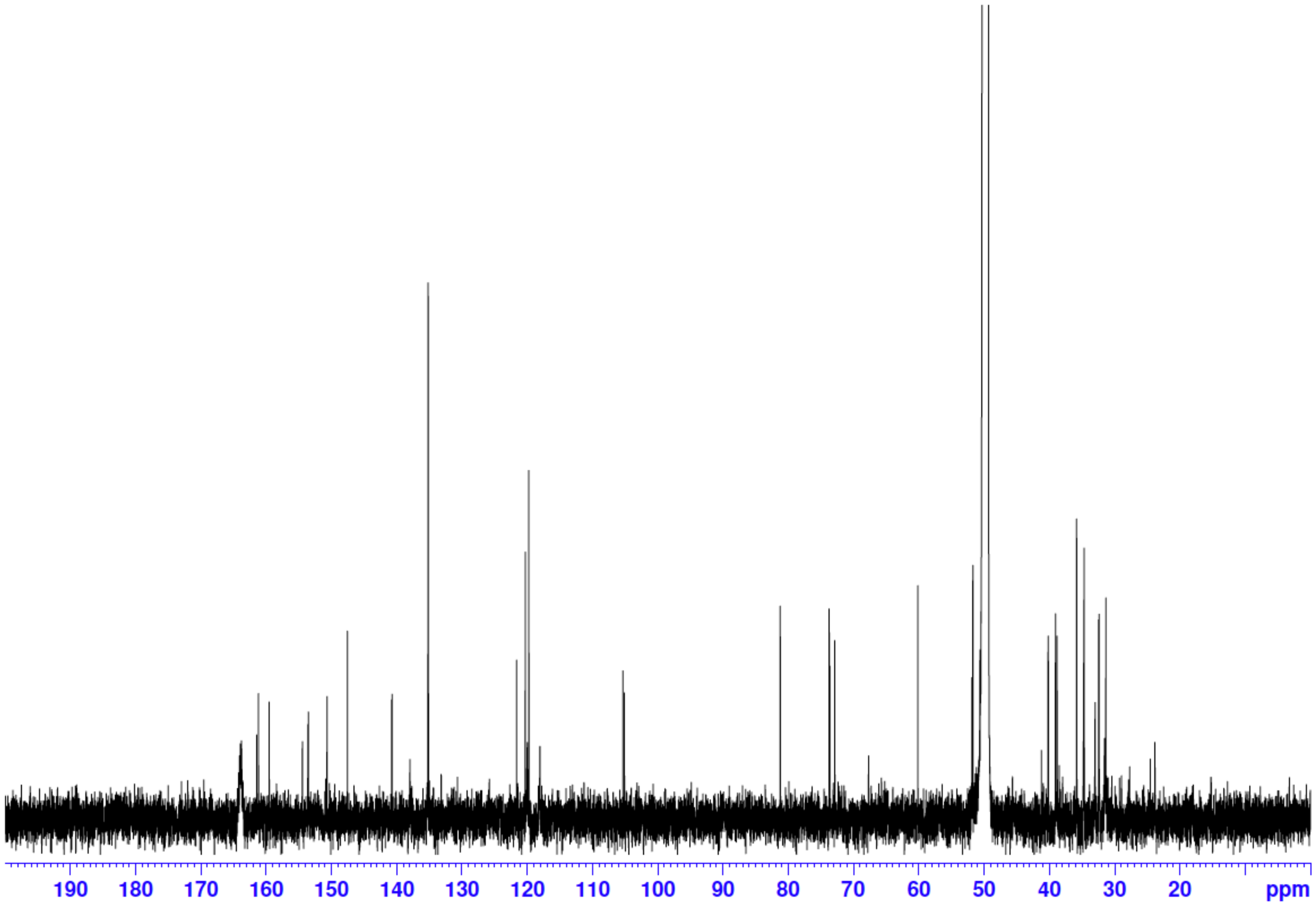

Figure S15. ^{1}H-^{1}H COSY spectrum of ceratinadin F (**2**) in CD_3OD (600 MHz).

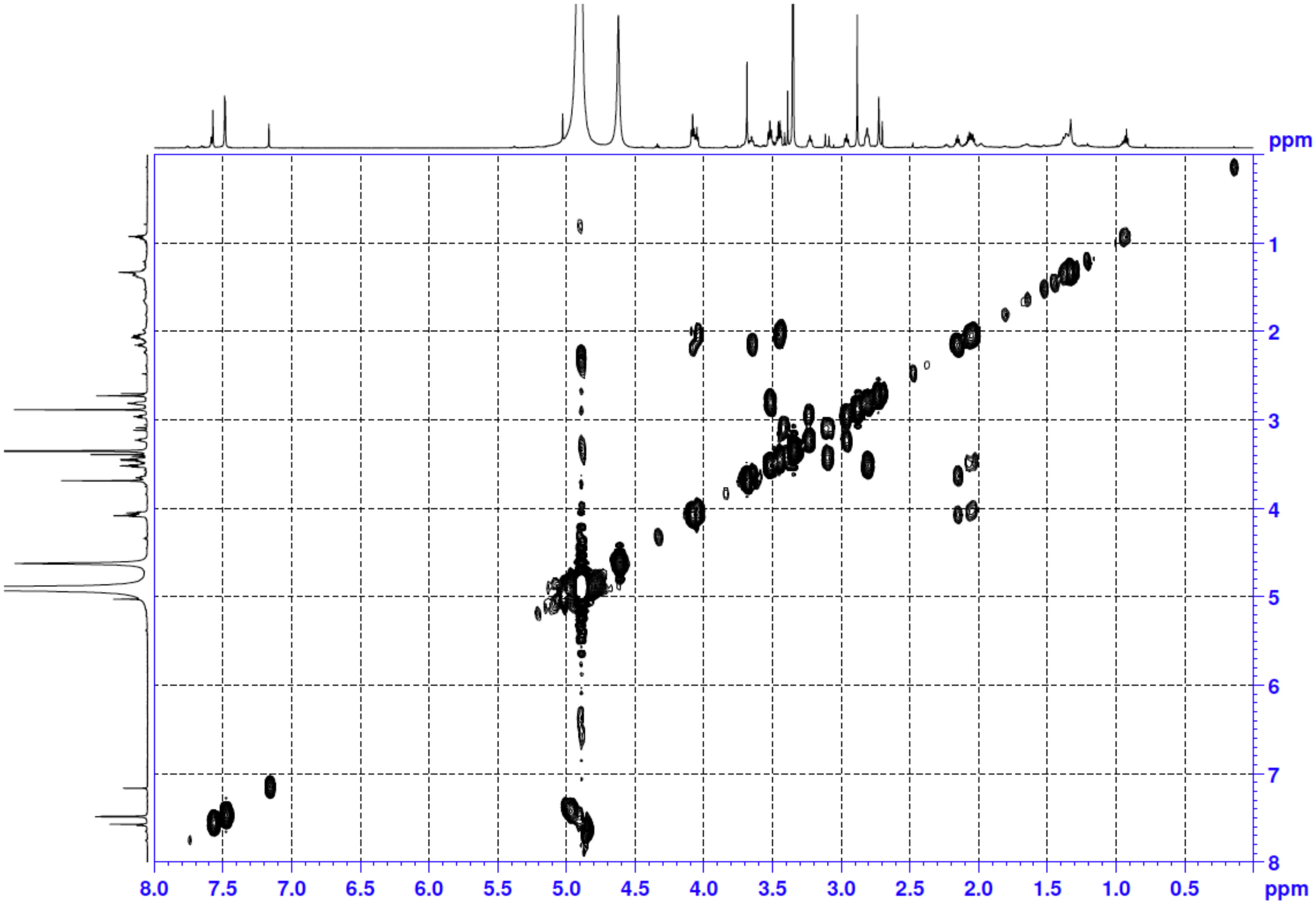

Figure S16. HSQC spectrum of ceratinadin F (**2**) in CD_3OD (600 MHz).

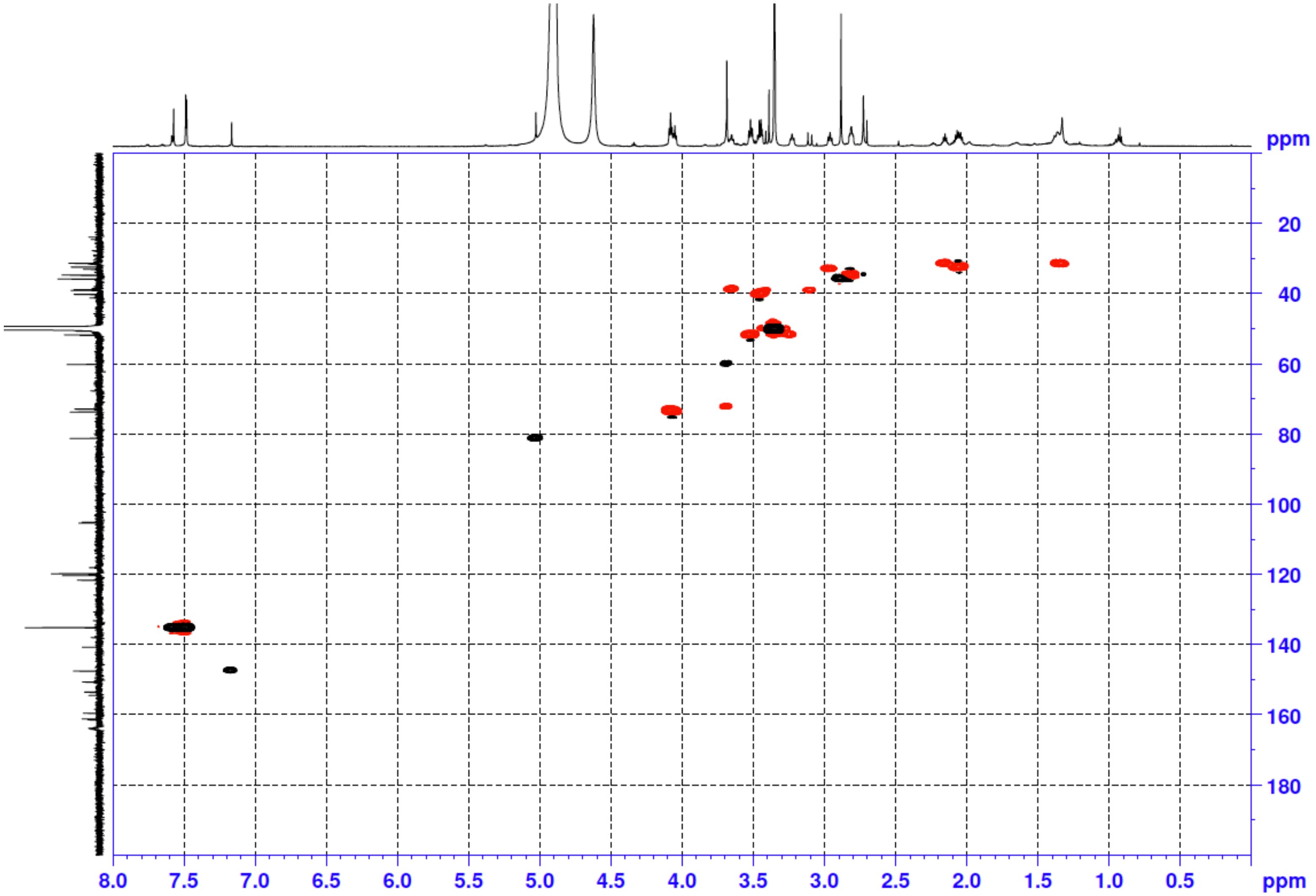

Figure S17. HMBC spectrum of ceratinadin F (**2**) in CD_3OD (600 MHz).

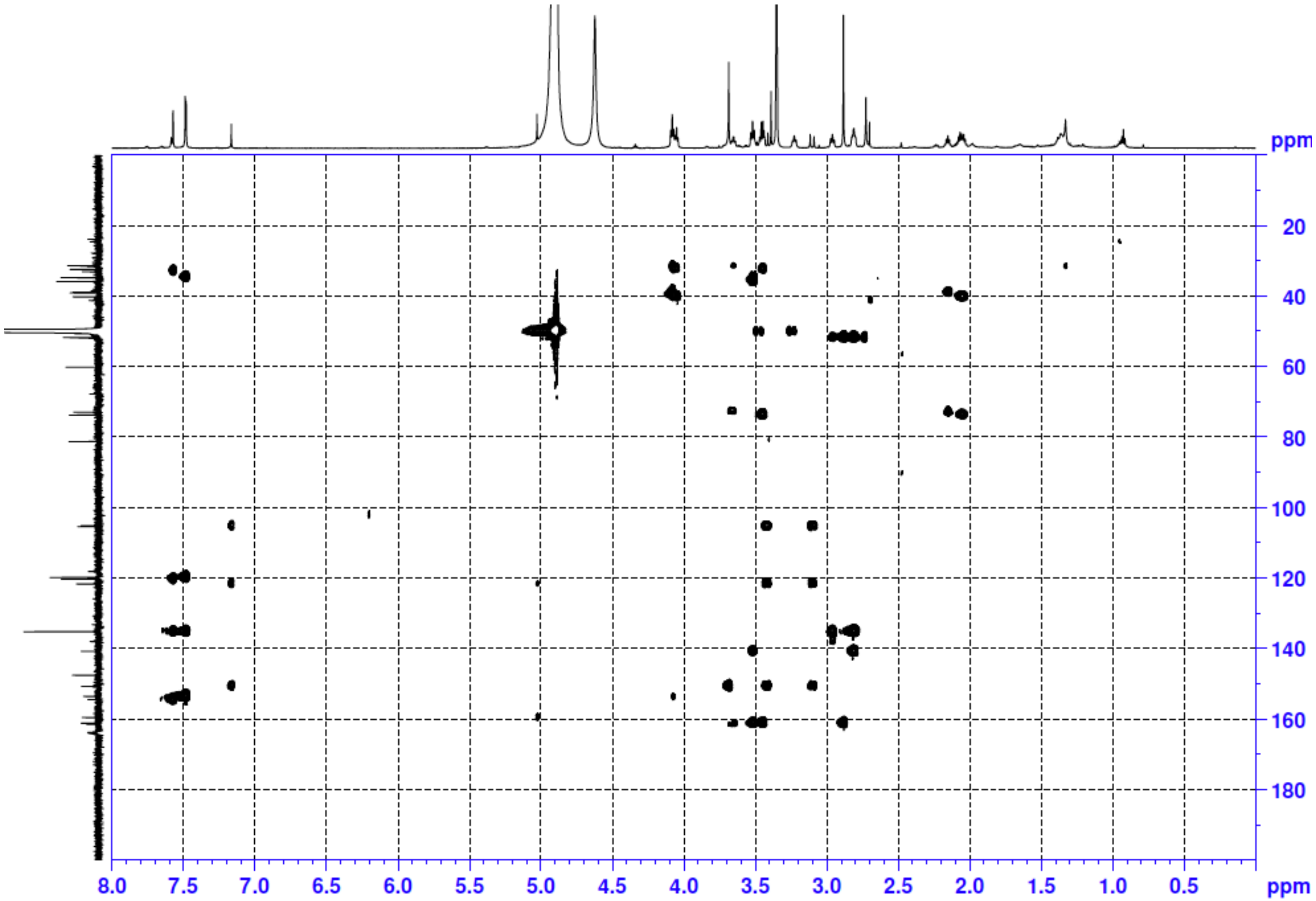

Figure S18. ECD spectrum of ceratinadin F (**2**) in CH_3OH.

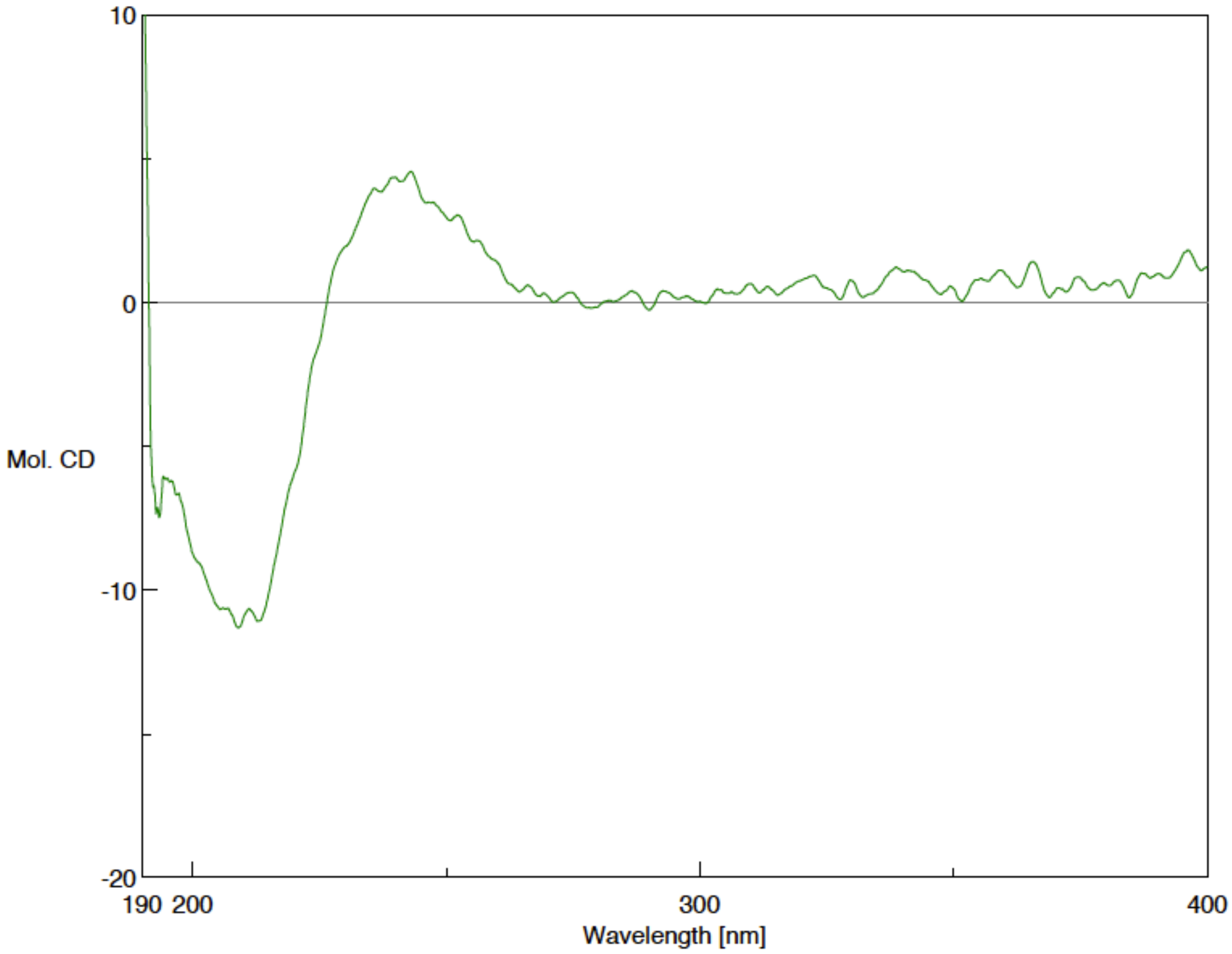

Supplement: Supplementary file 1 [file marinedrugs-16-00463-s001.pdf]
